# Effectiveness of Lactobacillus-supplemented eradication therapy for *Helicobacter pylori*: a systematic review and meta-analysis of randomized controlled trials

**DOI:** 10.1007/s00228-026-04186-5

**Published:** 2026-09-26

**Authors:** Karollyna M. Henry Moore, Paula Santo, Erica Uchoa Holanda, Angélica Luciana Nau, Gilmara Coelho Meine

**Affiliations:** 1 Fundación H. A. Barceló, Buenos Aires, Argentina; 2 Texas Tech University Health Sciences Center, Lubbock, TX USA; 3 University Center Christus, Fortaleza, Brazil; 4 Department of Pediatric Gastroenterology, Jaraguá Hospital, Jaraguá do Sul, Brazil; 5 Division of Gastroenterology, Department of Internal Medicine, Feevale University, Novo Hamburgo, Brazil

**Keywords:** *Helicobacter pylori*, *Lactobacillus*, Probiotics, Eradication, Meta-analysis

## Abstract

**Purpose:**

*Helicobacter pylori* eradication therapies supplemented with *Lactobacillus spp.* may improve eradication rates and reduce adverse events by modulating gut microbiota and immune responses. This meta-analysis aimed to evaluate the efficacy and safety of Lactobacillus-supplemented *Helicobacter pylori* eradication therapy.

**Methods:**

We systematically searched for randomized controlled trials (RCTs) comparing standard *Helicobacter pylori* eradication therapy with and without *Lactobacillus* in PubMed, Embase, and Cochrane Library up to August 2026. The primary outcome was eradication success, and the secondary outcome was adverse events. We calculated pooled risk ratios (RR) with 95% confidence intervals (CIs) using a random-effects model. We also estimated the number needed to treat (NNT) for eradication success and conducted subgroup analyses by antibiotic therapy type, duration of antibiotic treatment, Lactobacillus strain type, Lactobacillus dosage, period of Lactobacillus use, probiotic formulation, geographical region, and age range. This review was registered in PROSPERO (CRD42024611138).

**Results:**

Thirty-two RCTs (4,010 patients) were included. *Lactobacillus*-supplemented eradication therapy improved eradication success (RR 1.10; 95%CI 1.05–1.14; NNT 13) and reduced the risk of adverse events (RR 0.70; 95%CI 0.58–0.84). Subgroup analyses showed significant differences in eradication success by antibiotic regimen and in adverse event risk by age group. Improved eradication success with *Lactobacillus*-supplemented eradication therapy was observed only with triple therapy regimens, whereas adverse event risk was reduced only among adults.

**Conclusion:**

*Lactobacillus*-supplemented eradication therapy was associated with a 10% relative increase in *Helicobacter pylori* eradication success and a 30% relative reduction in treatment-related adverse events compared with the control group.

**Supplementary Information:**

The online version contains supplementary material available at https://doi.org/10.1007/s00228-026-04186-5.

## Introduction

*Helicobacter pylori* (*H. pylori*) infection affects nearly half of the global population and is a major cause of chronic gastritis, peptic ulcers, and gastric cancer [1, 2]. Standard therapies for *H. pylori* eradication face rising antibiotic resistance [3]. Therefore, it is essential to explore alternative or adjunctive strategies to optimize treatment outcomes.

Several studies suggested that probiotics, particularly *Lactobacillus spp*., administered alone or in conjunction with antibiotics, can improve eradication rates and reduce adverse events [4–6]. However, despite the promising results, uncertainty remains regarding the absolute clinical benefit of adjunctive *Lactobacillus*, its effect on overall treatment-related adverse events, and whether treatment effects vary across eradication regimens and supplementation strategies.

This meta-analysis aimed to evaluate the efficacy and safety of Lactobacillus supplementation as an adjunct to antibiotic eradication therapy against *H. pylori*, with subgroup analyses based on type of antibiotic therapy, duration of antibiotic treatment, type of *Lactobacillus* strain used, dosage of *Lactobacillus*, period of use of *Lactobacillus*, probiotic formulation, geographical region, and age range. Additionally, we conducted a meta-regression analysis to evaluate the impact of *Lactobacillus* dosage on the outcomes.

## Materials and methods

### Eligibility criteria

Inclusion in this meta-analysis was restricted to studies that met the following eligibility criteria: (1) randomized controlled trials (RCTs); (2) enrolling patients with confirmed *H. pylori* infection; (3) comparing triple or quadruple antibiotic *H. pylori* eradication therapy plus *Lactobacillus* with the same eradication therapy alone or with placebo; and (4) reporting at least one prespecified outcome of interest. Only original, full-text, peer-reviewed articles were included. No language restrictions were applied. We excluded studies (1) with observational design; (2) that used probiotic strains other than *Lactobacillus*; (3) using additional drugs beyond the antibiotic eradication therapy and *Lactobacillus*; or (4) probiotic monotherapy.

### Outcomes and additional analyses

The primary outcome was *H. pylori* eradication success, defined as the proportion of patients testing negative for H. pylori after completing therapy. The secondary outcome was overall adverse events associated with the treatment. We extracted outcome data from the intention-to-treat population.

We were uncertain regarding the modification of relative effects according to differences in baseline characteristics. Thus, to explain potential between-studies heterogeneity, we conducted subgroup analyses based on the following variables: (1) comparator type (placebo-controlled and non-placebo-controlled), (2) type of eradication regimen (standard triple [proton pump inhibitor/PPI + amoxicillin + clarithromycin], standard bismuth quadruple [PPI + bismuth + tetracycline + metronidazole], modified triple, modified bismuth quadruple, and other/sequential/unclassifiable regimens); (3) duration of antibiotic treatment (7–8 days; 10 days; and 14 days); (4) type of *Lactobacillus* strain (*Lactobacillus reuteri*; *Lactobacillus rhamnosus*; *Lactobacillus casei*; and *Lactobacillus acidophilus*); (5) dosage of *Lactobacillus* (< 1 × 10⁹ and ≥ 1 × 10⁹ colony-forming units [CFU]); (6) period of use of *Lactobacillus* (during antibiotic eradication therapy; during and after antibiotic eradication therapy; and before, during, and after antibiotic eradication therapy); (7) probiotic formulation (capsule; tablet; powder/sachet/packet; fermented milk/yogurt; and other not reported formulation); (8) geographical region (Europe; Asia; North America; and Africa); and (9) age range (children [< 18 years] and adults [≥ 18 years]). To further explore *Lactobacillus* dosage as a potential source of heterogeneity, we also conducted a random-effects meta-regression analysis using *Lactobacillus* dosage as the explanatory variable.

### Search strategy, study selection, and data extraction

We systematically searched PubMed, Embase, and the Cochrane Library databases from inception to August 2026. Supplementary Table  S1 describes the detailed search strategy for each database.

After retrieving the studies from the databases, two independent authors (K.M.H.M. and A.L.N.) excluded duplicates and screened titles and abstracts using Zotero Software (version 6.0.27; Digital Scholar; Fairfax, Virginia, USA). The eligibility of each remaining study was assessed based on the full-text review of the articles. We also searched for additional studies from references of the included RCTs and previous meta-analyses. A third author (G.C.M.) solved disagreements.

Two authors (K.M.H.M. and P.S.) independently extracted the following information from the included studies: general data (title, authors, country, and year of publication), sample size, patient characteristics (age and sex), characteristics of eradication therapy (antibiotics used, duration of antibiotic treatment, *Lactobacillus* strain used, dosage of *Lactobacillus*, period of use of *Lactobacillus*, and probiotic formulation), and outcomes of interest. We also extracted antimicrobial susceptibility and antibiotic-resistance data, including resistance to amoxicillin, clarithromycin, levofloxacin, and metronidazole, when reported.

### Quality assessment

Two authors (K.M.H.M. and E.U.H.) independently assessed the quality of included RCTs using the Cochrane Collaboration’s tool for assessing risk of bias in randomized trials (RoB2) [7], in which studies are scored as high, low, or unclear risk of bias across five domains: bias from the randomization process, bias due to deviations from intended interventions, bias due to missing outcome data, bias in measurement of outcomes, and bias in selection of the reported result. The assessment of each domain guided the overall judgment of the risk of bias. In cases of disagreement, the final decision was made through consensus. Publication bias was investigated by analyzing funnel plots of point estimates. The quality of evidence was evaluated using the Grading of Recommendations, Assessment, Development, and Evaluation (GRADE) tool. The GRADE system categorizes evidence quality into four levels: high, moderate, low, or very low certainty [8].

### Statistical analyses

The systematic review and meta-analysis were conducted in accordance with the Preferred Reporting Items for Systematic Reviews and Meta-Analyses (PRISMA) guidelines [9], and were registered in the Prospective Register of Systematic Reviews (PROSPERO) under the identification number CRD42024611138. We calculated the pooled risk ratios (RR) with 95% confidence intervals (CI) for binary outcomes using the Mantel-Haenszel method with a random-effects model, and a *P*-value < 0.05 was considered statistically significant. For eradication, we also pooled risk differences (RDs) using a random-effects model and derived the number needed to treat (NNT) from the pooled RD. We also conducted a proportion meta-analysis for both outcomes to calculate the pooled proportion of events in each group.

Between-study heterogeneity was assessed using Cochran’s Q test and quantified using the I² statistic. A P-value < 0.10 for Cochran’s Q test was considered indicative of statistically significant heterogeneity, and I² values of ≤ 25%, > 25% to 50%, and > 50% were interpreted as indicating low, moderate, and high heterogeneity, respectively. When heterogeneity was at least moderate, we performed a leave-one-out sensitivity analysis. We conducted prespecified subgroup analyses if there were at least two studies in each subgroup, and a *P*-value < 0.05 was regarded as statistically significant when assessing subgroup differences. The meta-regression analysis by *Lactobacillus* dosage was performed if there were at least 10 studies available, meeting the threshold for reliable analysis. We used Review Manager 5.3 (Cochrane Centre, The Cochrane Collaboration, Denmark) for pair-wise meta-analysis and subgroup analysis, and R statistical software (version 4.2.1; R Foundation for Statistical Computing, Vienna, Austria) for proportion meta-analysis and random-effects meta-regression on the risk-difference scale when at least 10 studies had usable data [10].

## Results

### Study selection and characteristics

**Fig. 1:**
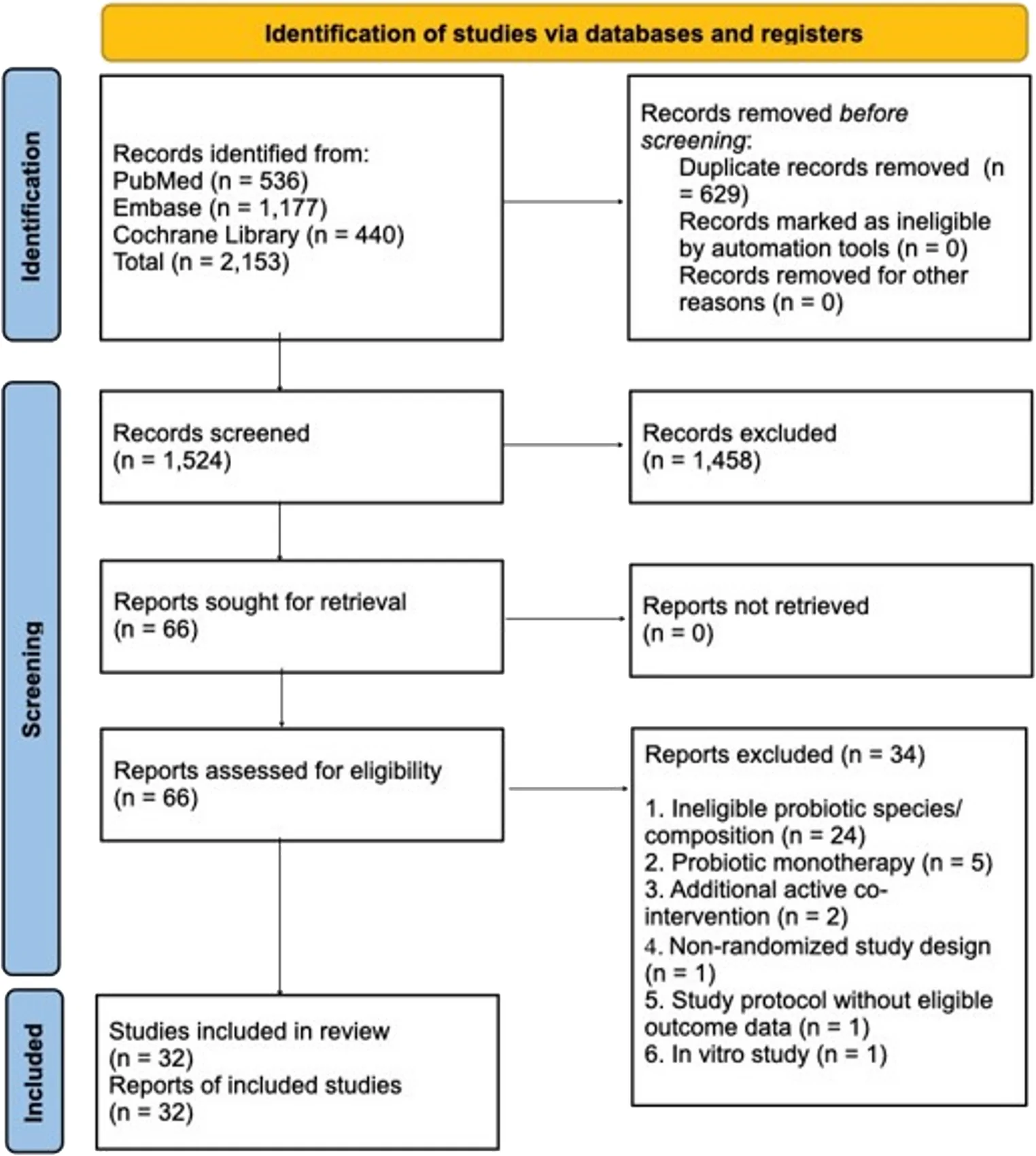
Preferred Reporting Items for Systematic Reviews and Meta-Analyses (PRISMA) flow diagram of study screening and selection

As shown in Fig. 1, the initial search yielded 2,153 results. After removing duplicates and excluding ineligible studies, we fully reviewed 66 studies against the inclusion criteria. Ultimately, we included 32 RCTs in this meta-analysis [11–42], comprising 4,010 patients (1,935 participants received antibiotic eradication therapy supplemented with Lactobacillus, and 2,075 patients received antibiotic eradication therapy without Lactobacillus). Poonyam et al. [11] contributed separate 7-day and 14-day comparisons. Comparator design varied across the included trials. In some studies, participants in the control group received eradication therapy supplemented with placebo [11, 12, 15, 19–24, 26–31, 37, 38, 40, 41], whereas in others, the control group received eradication therapy without Lactobacillus supplementation [13, 14, 16–18, 25, 32–36, 39, 42]. Supplementary Table S2 lists the excluded studies at the full-text screening stage and the reasons for exclusion. A detailed description of the baseline characteristics of the included studies, including patient demographics and intervention specifics, is provided in Tables 1 and 2, and Supplementary Table S3.

**Table 1:** Baseline characteristics of included studies: Study and participant characteristics

First author, year	Country	Population	Sample size I/C	Age, mean (SD) I/C	Male, *n* (%) overall
Armuzzi [12] (APT)	Italy	Adults	30/30	Overall: 40 (12)	25/60 (41.7%)
Armuzzi [13] (Digestion)	Italy	Adults	60/60	37.0 (11.3)/36.8 (10.0)	54/120 (45.0%)
Canducci [14]	Italy	Adults	60/60	Overall: 38 (11)	64/120 (53.3%)
Cremonini [15]	Italy	Adults	21/21	N/R	N/R
da Silva Medeiros [16]	Portugal	Adults	31/31	52.0 (22–80)/58.5 (24–78)*	32/62 (51.6%)
Deguchi [17]	Japan	Adults	115/114	55.9/57.8	142/229 (62.0%)
Dore [18]	Italy	Adults	49/50	54.1 (14)/52.2 (14)	29/99 (29.3%)
Emara [19]	Egypt	Adults	35/35	33.20 (13.97)/36.80 (11.09)	24/70 (34.3%)
Francavilla [20]	Italy	Adults	50/50	49 (22–65)/44 (18–61)*	39/100 (39.0%)
Fu [21]	China	Adults	36/36	45.38 (12.16)/41.74 (11.29)	25/68 (36.8%)
Han [22]	China	Adults	128/129	39.99 (8.46)/40.79 (7.95)	0/257 (0%)
Ismail [23]	Malaysia	Adults	45/45	49.0 (37.5–68.8)/53.0 (37.0–68.5)*	38/89 (42.7%)
Ivashkin [24]	Russia	Adults	66/63	47 (38–56)/47 (36–53)*	40/117 (34.2%)
Kamani [25]	Pakistan	Adults	195/195	36.51 (9.91)/53.65 (10.0)	178/390 (45.6%)
Kiattiweerasak [26]	Thailand	Adults	45/45	54.5 (10.5)/55.3 (9.5)	43/90 (47.8%)
Lionetti [27]	Italy	Children	20/20	11.0 (3.3–18)/9.9 (4.3–17.6)*	21/40 (52.5%)
Ma [28]	China	Adults	66/66	38.6 (7.8)/37.8 (8.2)	69/132 (52.3%)
Mohtasham [29]	Iran	Participants aged > 9 years	225/225	53.05 (12.01)/52.72 (14.40)	206/450 (45.8%)
Moreno Márquez [30]	Spain	Adults	40/40	49.4 (17.0)/51.6 (18.0)*	38/80 (47.5%)
Niu [31]	China	Adults	36/36	48.03 (9.61)/45.23 (11.95)	24/71 (33.8%)
Ojetti [32]	Italy	Adults	45/45	41.5 (11.7)/43.1 (13.3)	60/90 (66.7%)
Paoluzi [33]	Italy	Adults	71/71	Overall: 53.0 (12.7)	43/142 (30.3%)
Parth [34]	India	Adults	45/45	N/R	52/90 (57.8%)
Poonyam [11] (A)	Thailand	Adults	25/25	7-day groups overall: 56	17/50 (34.0%)
Poonyam [11] (B)	Thailand	Adults	25/25	14-day groups overall: 52	11/50 (22.0%)
Sahagún-Flores [35]	Mexico	Adults	35/36	45.2 (8.8)/45.3 (7.5)	37/71 (52.1%)
Shahraki [36]	Iran	Children	25/25	8.5 (2.5)/7.8 (2.5)	17/50 (34.0%)
Sýkora [37]	Czech Republic	Children	39/47	12.6 (3.3)/12.9 (3.7)	34/86 (39.5%)
Szajewska [38]	Poland	Children	44/39	12.3 (2.7)/11.9 (3.1)	39/83 (47.0%)
Tursi [39]	Italy	Adults	35/35	58.2 (27–71)/54.3 (31–66)**	40/70 (57.1%)
Yang [40]	China	Adults	100/100	N/R	N/R
Zhao [41]	China	Adults	47/47	43.43 (11.63)/43.85 (12.02)	30/94 (31.9%)
Ziemniak [42]	Poland	Adults	53/192	44.4 (13.3)/43.7 (10.3)	92/245 (37.6%)

* median (range or interquartile range); ** mean (range). *C* comparator, *I* Lactobacillus intervention, *N/R* not reported, *SD *= standard deviation

**Table 2:** Baseline characteristics of included studies: Comparator, eradication regimen, and antibiotic resistance

First author, year	Comparator	Antibiotic regimen class	Antibiotic eradication regimen	Antibiotic resistance reporting
Armuzzi [12] (APT)	Placebo-controlled	Modified triple	7 d: rabeprazole 20 mg bid + clarithromycin 500 mg bid + tinidazole 500 mg bid.	N/A
Armuzzi [13] (Digestion)	Eradication therapy alone	Modified triple	7 d: pantoprazole 40 mg bid + clarithromycin 500 mg bid + tinidazole 500 mg bid.	N/A
Canducci [14]	Eradication therapy alone	Standard triple	7 d: rabeprazole 20 mg bid + clarithromycin 250 mg tid + amoxicillin 500 mg tid.	N/A
Cremonini [15]	Placebo-controlled	Modified triple	7 d: rabeprazole 20 mg bid + clarithromycin 500 mg bid + tinidazole 500 mg bid.	N/A
da Silva Medeiros [16]	Eradication therapy alone	Standard triple	8 d: esomeprazole 20 mg bid + amoxicillin 1 g bid + clarithromycin 500 mg bid.	AST performed in 59/62 patients. Resistance prevalence: N/A.
Deguchi [17]	Eradication therapy alone	Standard triple	7 d: rabeprazole 10 mg bid + amoxicillin 750 mg bid + clarithromycin 200 mg bid.	CAM resistance: 27.1%.
Dore [18]	Eradication therapy alone	Modified triple ^b^	10 d: pantoprazole 20 mg bid + tetracycline 500 mg bid + metronidazole 500 mg bid	N/A
Emara [19]	Placebo-controlled	Standard triple	14 d: omeprazole 20 mg bid + amoxicillin 1 g bid + clarithromycin 500 mg bid.	N/A
Francavilla [20]	Placebo-controlled	Standard triple	7 d: PPI + clarithromycin + amoxicillin; exact doses not reported.	N/A
Fu [21]	Placebo-controlled	Bismuth quadruple	N/A	N/A
Han [22]	Placebo-controlled	Modified bismuth quadruple	14 d: rabeprazole 10 mg bid + potassium bismuth citrate 220 mg bid + amoxicillin 1 g bid + furazolidone 100 mg bid.	N/A
Ismail [23]	Placebo-controlled	Standard triple ^a^	14 d: esomeprazole 40 mg bid + amoxicillin 1 g bid + clarithromycin 500 mg bid	N/A
Ivashkin [24]	Placebo-controlled	Standard triple	14 d: esomeprazole 20 mg bid + amoxicillin 1 g bid + clarithromycin 500 mg bid.	N/A
Kamani [25]	Eradication therapy alone	Other	Sequential regimen: esomeprazole 20 mg bid for 14 d; amoxicillin 1 g bid on days 1–5; clarithromycin 500 mg bid + tinidazole 500 mg bid on days 6–10.	N/A
Kiattiweerasak [26]	Placebo-controlled	Standard triple	14 d: lansoprazole 30 mg bid + amoxicillin 1 g bid + clarithromycin modified-release 1 g qd.	CAM resistance: 24%; AMX resistance: 0%.
Lionetti [27]	Placebo-controlled	Other	Days 1–5: omeprazole 1 mg/kg/day + amoxicillin 50 mg/kg/day. Days 6–10: omeprazole 1 mg/kg/day + clarithromycin 15 mg/kg/day + tinidazole 20 mg/kg/day.	N/A
Ma [28]	Placebo-controlled	Modified triple	7 d: omeprazole 20 mg qd + clarithromycin 500 mg bid + metronidazole 400 mg tid.	N/A
Mohtasham [29]	Placebo-controlled	Modified bismuth quadruple	14 d: bismuth subcitrate 240 mg bid + pantoprazole 40 mg bid + amoxicillin 1 g bid + clarithromycin 500 mg bid.	N/A
Moreno Márquez [30]	Placebo-controlled	Standard bismuth quadruple	10 d: three-in-one capsules (bismuth subcitrate potassium 140 mg + metronidazole 125 mg + tetracycline 125 mg), 3 capsules qid + omeprazole 40 mg bid.	N/A
Niu [31]	Placebo-controlled	Modified bismuth quadruple	14 d: rabeprazole 20 mg bid + levofloxacin 250 mg bid + metronidazole 400 mg bid + bismuth pectin 200 mg tid.	N/A
Ojetti [32]	Eradication therapy alone	Modified triple	7 d: esomeprazole 20 mg bid + levofloxacin 500 mg bid + amoxicillin 1 g bid.	N/A
Paoluzi [33]	Eradication therapy alone	Modified triple	7 d: esomeprazole 20 mg bid + levofloxacin 500 mg bid + doxycycline 200 mg bid.	Reported AST in 39 patients. LVX resistance: 18%.
Parth [34]	Eradication therapy alone	Standard triple	14 d: esomeprazole 40 mg bid + clarithromycin 500 mg bid + amoxicillin 1 g bid.	N/A
Poonyam [11] (A)	Placebo-controlled	Standard bismuth quadruple	7 d: dexlansoprazole 60 mg bid + bismuth subsalicylate 1,048 mg bid + metronidazole 400 mg tid + tetracycline 500 mg qid.	Reported AST in 68% of included patients (data refer to the overall study population). MTZ resistance: 34.1%.
Poonyam [11] (B)	Placebo-controlled	Standard bismuth quadruple	14 d: dexlansoprazole 60 mg bid + bismuth subsalicylate 1,048 mg bid + metronidazole 400 mg tid + tetracycline 500 mg qid.	Reported AST in 68% of included patients (data refer to the overall study population). MTZ resistance: 34.1%.
Sahagún-Flores [35]	Eradication therapy alone	Standard triple	7 d: omeprazole 20 mg bid + amoxicillin 1 g bid + clarithromycin 500 mg bid.; omeprazole continued to 28 d total.	N/A
Shahraki [36]	Eradication therapy alone	Standard triple	14 d: amoxicillin 50 mg/kg/day + clarithromycin 15 mg/kg/day in divided doses; omeprazole 1 mg/kg/day in 2 divided doses for 1 mo.	N/A
Sýkora [37]	Placebo-controlled	Standard triple	7 d: omeprazole 10 mg bid (15–30 kg) or 20 mg bid (> 30 kg) + amoxicillin 25 mg/kg bid + clarithromycin 7.5 mg/kg bid.	CAM resistance: 21.2%
Szajewska [38]	Placebo-controlled	Standard triple	7 d: omeprazole 0.5 mg/kg bid + amoxicillin 25 mg/kg bid (max 1.5 g/day) + clarithromycin 10 mg/kg bid (max 1 g/day).	N/A
Tursi [38]	Eradication therapy alone	Modified bismuth quadruple	10 d: ranitidine bismuth citrate 400 mg bid + esomeprazole or pantoprazole 40 mg qd + amoxicillin 1 g tid + tinidazole 500 mg bid.; PPI continued for 4 wk in patients with active ulcer or severe gastritis.	N/A
Yang [40]	Placebo-controlled	Standard triple	14 d: esomeprazole 20 mg bid + amoxicillin 1 g bid + clarithromycin 500 mg bid.	N/A
Zhao [41]	Placebo-controlled	Modified bismuth quadruple	14 d: omeprazole 20 mg bid + colloidal bismuth pectin 200 mg bid + amoxicillin 1 g bid + clarithromycin 500 mg bid.	N/A
Ziemniak [42]	Eradication therapy alone	Standard triple	10 d: pantoprazole 40 mg bid + clarithromycin 500 mg bid + amoxicillin 1 g bid.	N/A

^a^ metronidazole 400 mg bid replaced amoxicillin in penicillin-allergic patients; ^b^ The control group also received bismuth subcitrate potassium 280 mg bid. *AMX* amoxicillin, *AST* antimicrobial susceptibility testing, *bid* twice daily, *CAM* clarithromycin, *LVX* levofloxacin, *MTZ* metronidazole, *N/A* not available, *qd* once daily, *qid* four times daily, *tid* three times daily

### Pooled analyses

#### Eradication success

**Fig. 2:**
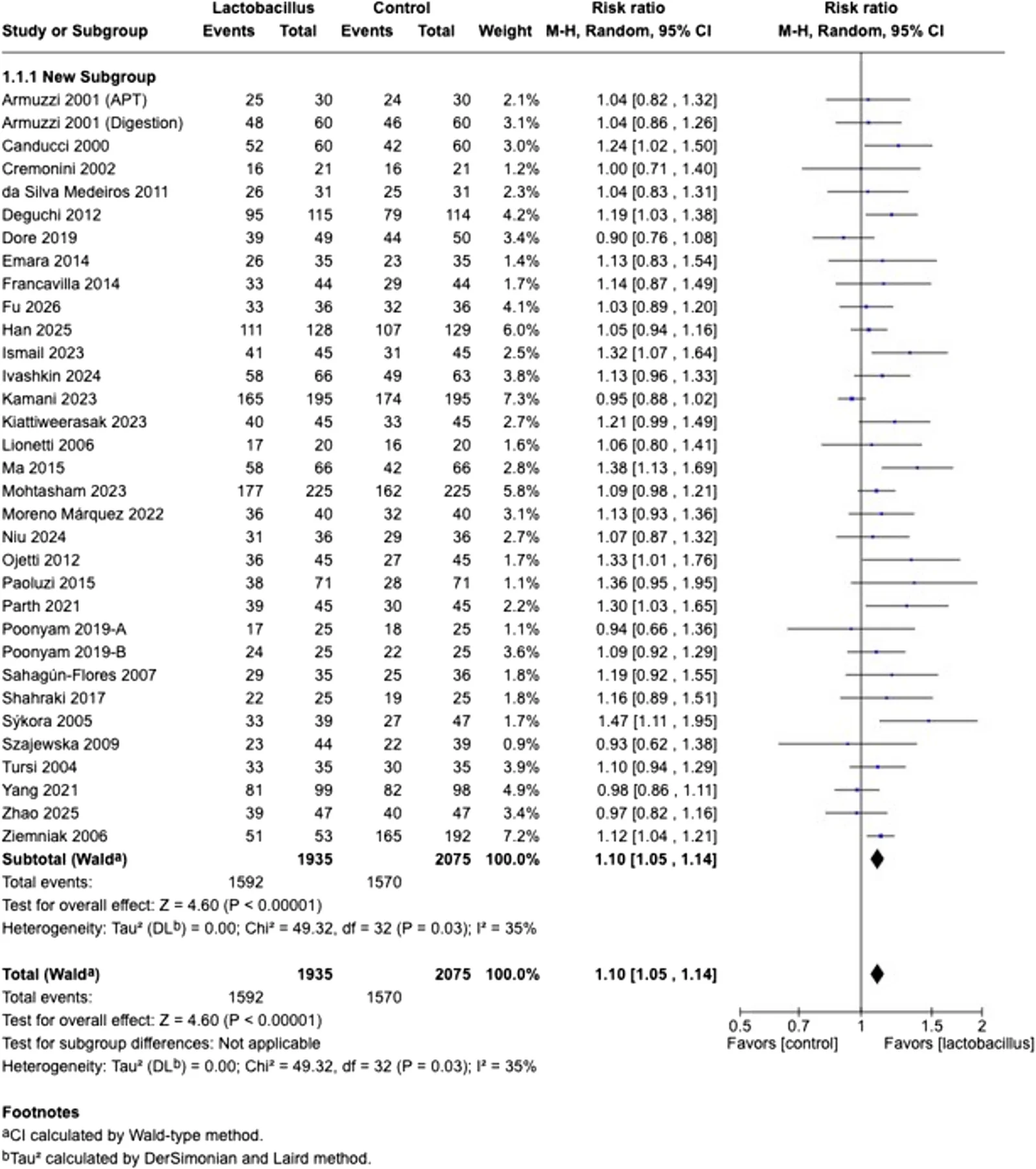
Forest plot of *Helicobacter pylori* eradication success. CI, confidence interval; M-H, Mantel–Haenszel

All included RCTs reported the *H. pylori* eradication rate. The pooled eradication success rate in the *Lactobacillus*-supplemented therapy group was 83.64% (95% CI 80.34%–86.48%), compared with 75.55% (95% CI 71.41%–79.26%) in the control group. The probability of successful eradication was significantly higher with *Lactobacillus*-supplemented therapy (RR 1.10; 95% CI 1.05–1.14; *P* < 0.00001; I²=35%; Fig. 2). The RD was 0.08 (95% CI 0.05–0.11), corresponding to NNT 13 (95% CI 10–20). In the leave-one-out sensitivity analysis, the exclusion of the study by Kamani et al. [25] reduced between-study heterogeneity and did not significantly impact the effect estimate (RR 1.11; 95% CI 1.07–1.15; I²=14%; Supplementary Table S4).

#### Overall adverse events

**Fig. 3:**
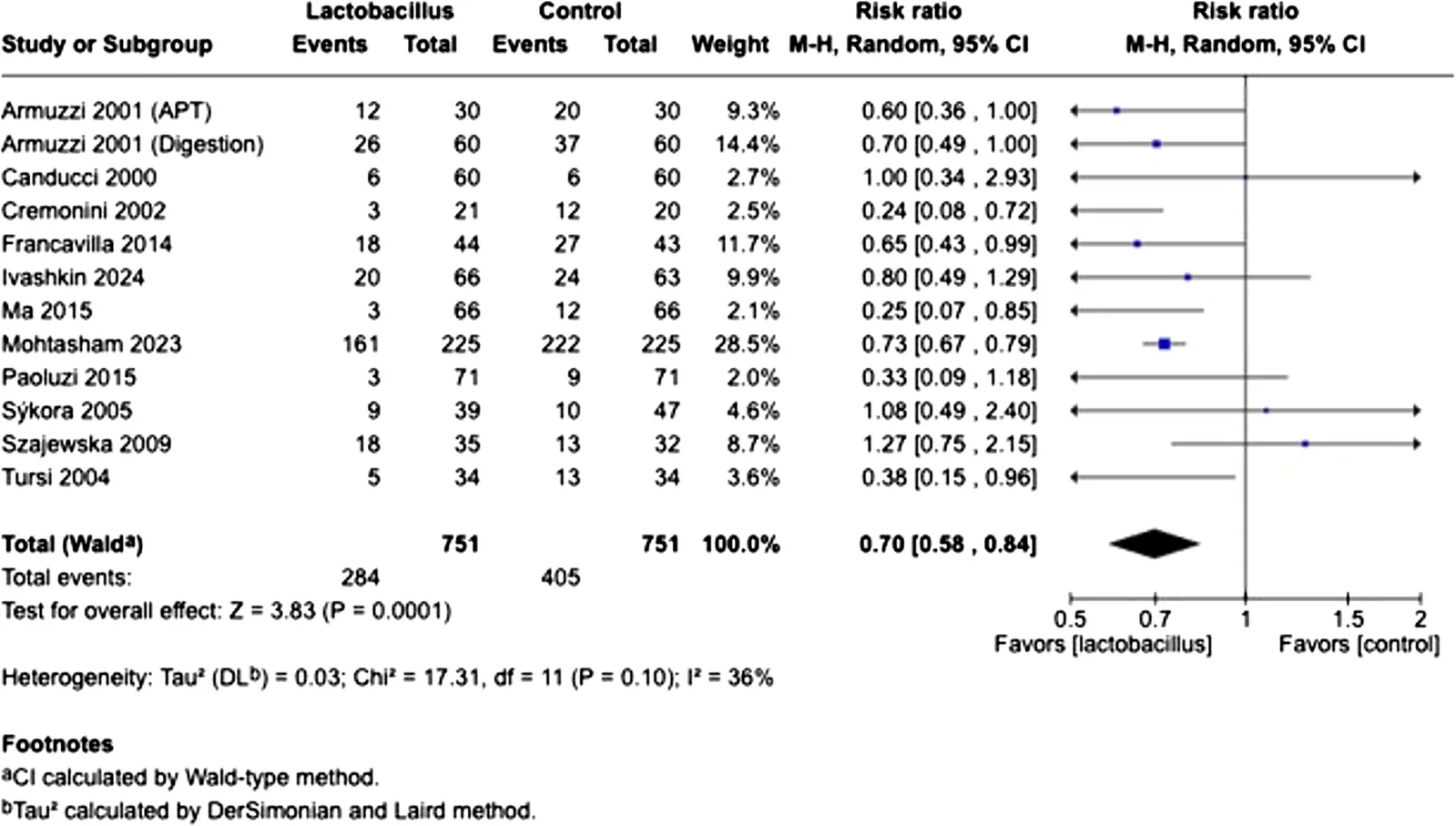
Forest plot of overall adverse events. CI, confidence interval; M-H, Mantel–Haenszel

Twelve RCTs reported adverse event rates. The *Lactobacillus*-supplemented therapy group had a pooled adverse events rate of 24.27% (95% CI 13.80%–39.07%), compared to 45.54% (95% CI 24.89%–67.84%) in the standard therapies group. *Lactobacillus*-supplemented therapy significantly reduced the risk of overall adverse events (RR 0.70; 95% CI 0.58–0.84; *P* = 0.0001; I²=36%; Fig. 3). In the leave-one-out sensitivity analysis, excluding the study by Cremonini et al. [15] reduced between-study heterogeneity and did not significantly affect the effect estimate (RR 0.72; 95% CI 0.62–0.84; I²=23%; Supplementary Table S5). Regarding individual adverse events, *Lactobacillus*-supplemented therapy reduced the risk of heartburn (RR 0.32; 95% CI 0.22–0.47), nausea (RR 0.48; 95% CI 0.24–0.98), and diarrhea (RR 0.36; 95% CI 0.24–0.55), but did not significantly impact abdominal pain (RR 0.62; 95% CI 0.37–1.03; Supplementary Figure S1).

### Additional analyses

Subgroup analyses of *H. pylori* eradication success are presented in Supplementary Figures S2–S10. Subgroup analysis according to eradication regimen revealed a statistically significant between-subgroup difference (P for subgroup difference = 0.0004). Lactobacillus supplementation showed a significant benefit for standard triple therapy (RR 1.14; 95% CI 1.09–1.20; I² = 10%) and modified triple therapy (RR 1.18; 95% CI 1.04–1.34; I² = 34%) but not for the other regimen classes. Supplementary Figures S11–S18 present subgroup analyses of adverse events. We observed a statistically significant between-subgroup difference by age group (P for subgroup difference = 0.007). *Lactobacillus* supplementation was associated with a significant reduction in adverse events among adults (RR 0.61; 95% CI 0.48–0.77; I² = 17%) but not among children. However, this finding should be considered exploratory because data for the pediatric subgroup were limited. No statistically significant between-subgroup differences were detected in any of the other subgroup analyses of eradication success or adverse events.

Meta-regression analyses showed no significant association between *Lactobacillus* dosage and the effect estimates for either eradication success or adverse events (Supplementary Table S6).

Six studies reported antibiotic susceptibility. Clarithromycin resistance ranged from 15.6% to 27.1%, whereas metronidazole and levofloxacin resistance were 34.1% and 18%, respectively, in one study each. No amoxicillin resistance was detected in the studies reporting this outcome. In da Silva Medeiros et al. [16], all 11 treatment failures involved strains susceptible to both clarithromycin and amoxicillin. Among patients with clarithromycin-resistant strains, Deguchi et al. [17] and Kiattiweerasak et al. [26] found no statistically significant difference in eradication between the *Lactobacillus* and control groups, whereas Sýkora et al. [37] reported rates of 83% and 25%, respectively. Among metronidazole-resistant strains, Poonyam et al. [11] reported eradication rates with versus without *L. reuteri* of 100% (2/2) versus 50% (2/4) with the 7-day regimen and 100% (4/4) versus 75% (3/4) with the 14-day regimen.

### Quality assessment

The risk of bias assessment of individual RCTs using the Cochrane RoB2 tool indicated that most studies had some concerns and three studies were judged to be at high overall risk of bias: Paoluzi et al. [33] and da Silva Medeiros et al. [16], because of the randomization process; and Ziemniak [42], because of substantial missing outcome data (Supplementary Figure S19).

**Fig. 4:**
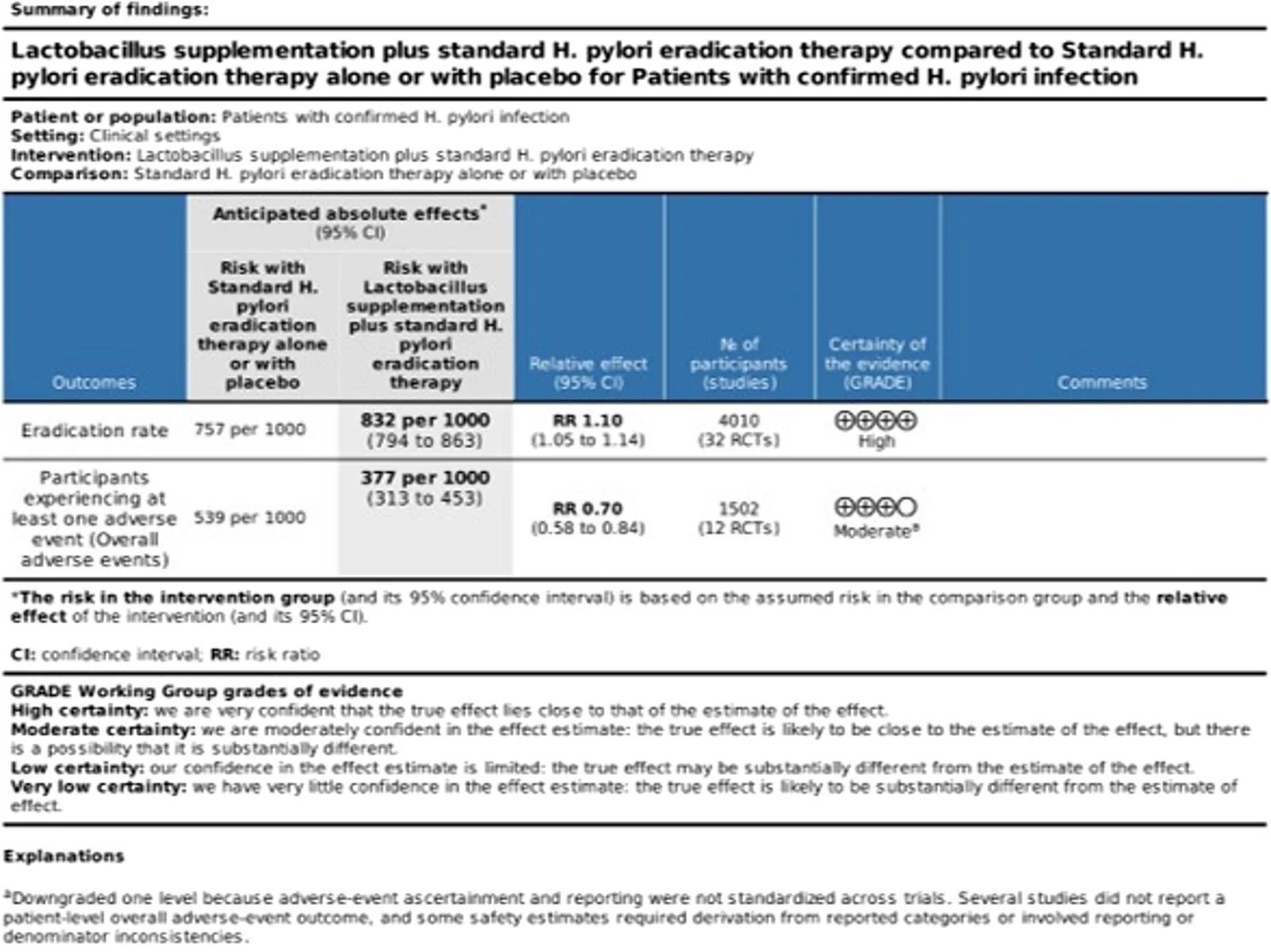
Summary of Findings for *Helicobacter pylori* eradication success and overall adverse events

Funnel plot analysis of eradication success suggests asymmetry, with a relative paucity of small studies reporting null or unfavorable effects, raising concern for small study effects and possible publication bias. For overall adverse events, visual inspection of the funnel plot suggested asymmetry, but interpretation was limited by the small number of informative studies, sparse events, and studies falling outside the displayed axis range. Therefore, publication bias could not be reliably assessed (Supplementary Figures S20 and S21). Figure 4 shows the summary of findings and the certainty of evidence assessment according to the GRADE tool.

## Discussion

This meta-analysis, which included 32 RCTs, involving 4,010 patients across diverse populations, provided evidence supporting the use of *Lactobacillus* supplementation as an adjunct therapy in *H. pylori* eradication therapy. We observed that *Lactobacillus* supplementation significantly improved eradication success and reduced the risk of adverse events compared to standard antibiotic therapy.

The increasing interest in probiotics for *H. pylori* eradication stems from challenges associated with antibiotic resistance, particularly biofilm formation and efflux pump mechanisms, which reduce antibiotic efficacy, and nutrient transporters in *H. pylori* that may contribute to resistance [43, 44]. Additionally, antibiotic misuse further exacerbates resistance issues [3].

*Lactobacillus* supplementation improves *H. pylori* eradication through multiple mechanisms. Probiotics enhance the gut’s innate defenses by modulating interactions between *H. pylori* and the gastric mucosa. Certain *Lactobacillus* strains increase mucin expression, counteracting the suppression typically induced by *H. pylori* infection. Additionally, some strains exhibit anti-inflammatory properties, reducing the production of pro-inflammatory cytokines. Probiotics also strengthen the gastric immunologic barrier by stimulating local immunoglobulin A responses and alleviating inflammation, helping to stabilize the gastric mucosa. Furthermore, strains like *L. reuteri* produce antimicrobial compounds such as reuterin, which exert both bactericidal and bacteriostatic effects against a wide range of pathogens [45].

Our findings support the efficacy of *Lactobacillus* supplementation as an adjunct therapy for *H. pylori* eradication, consistent with previous meta-analyses [46–49]. However, there were important methodological differences between these studies and ours that may explain the differences in the magnitude of the effects and safety findings. Li et al. [46] focused exclusively on *L. reuteri* supplementation, reporting improved eradication rates and reduced overall adverse events. Yu et al. [47] included a smaller sample size and restricted the analysis to *Lactobacillus*-supplemented triple therapy; although they found improved eradication rates, they reported similar overall adverse event rates between groups, with significant benefit only for taste disturbance. Mishra et al. [48] reported improved eradication and reductions in selected gastrointestinal adverse events, but included studies that did not meet our eligibility criteria, such as observational design (Marinelli et al. [49]), antibiotic monotherapy without PPI (Felley et al. [50]), a trial in which *Lactobacillus* replaced rather than supplemented one component of the eradication regimen (Dore et al. [51]), and *Lactobacillus* not administered concomitantly with eradication therapy (Francavilla et al. [52]). Additionally, their subgroup analyses did not specify whether subgroup differences were statistically significant. More recently, Azam et al. [53] included 14 randomized controlled trials, several of which evaluated multi-species formulations that also contained non-*Lactobacillus* probiotics.

This review updates the evidence by including 32 studies and extends previous findings by quantifying the absolute eradication benefit, pooling overall adverse events, and examining effects across comparator designs and a broader range of treatment and supplementation characteristics. Although the studies included in our analyses varied in methodology, our subgroup analyses identified factors associated with optimized outcomes. However, these findings should be considered exploratory and interpreted cautiously, particularly the age-based findings because pediatric data were limited.

Evidence on how antibiotic resistance influenced Lactobacillus supplementation outcomes was limited and inconsistent. Two studies reported no statistically significant between-group difference among patients with clarithromycin-resistant strains, whereas Sýkora et al. [37] and Poonyam et al. [11] reported numerically higher eradication rates with *Lactobacillus* in resistant infections. However, susceptibility testing was available only for subsets of participants, the resistant subgroups were very small, and formal treatment-by-resistance interaction analyses were not performed. Moreover, all failures in da Silva Medeiros et al. [16] occurred despite in vitro susceptibility, suggesting that factors beyond antimicrobial resistance may contribute to treatment failure. Therefore, the available evidence does not allow us to determine whether *Lactobacillus* supplementation overcomes antibiotic resistance or preferentially benefits patients harboring resistant strains.

Other emerging approaches to enhance *H. pylori* eradication include less conventional strategies, such as oral vaccination. Probiotics, particularly *L. lactis*, have shown promise as a vaccine delivery system, mitigating the challenges posed by acidic gastric conditions that typically reduce vaccine efficacy [54]. Additionally, new *H. pylori* eradication alternatives, including vonoprazan-based therapies and rifabutin, show promising results [55].

The present study has some strengths. We included only RCTs, minimizing the risk of confounding factors typically associated with observational studies. Additionally, the large sample size (4,010 patients) and rigorous prespecified methods further strengthen the reliability and robustness of our findings.

However, our meta-analysis has several limitations. First, most studies were conducted in Europe and Asia, limiting the generalizability of the findings to other continents. Second, pediatric evidence was limited, comprising 259 participants in the eradication analysis and 153 participants with available adverse-event data, which limited statistical power and precluded firm conclusions for this population. Third, comparator design varied across trials. Some studies used placebo-controlled designs, whereas others compared Lactobacillus supplementation with eradication therapy alone. Fourth, because data were insufficient, we could not assess symptom improvement using the Gastrointestinal Symptom Rating Scale or evaluate adverse-event severity. Fifth, antibiotic-resistance data were inconsistently reported, and resistance-stratified eradication outcomes were too sparse for reliable pooled analysis. Sixth, only 12 of the 32 included studies provided consistent binary data for overall adverse events. Finally, methodological differences among the studies may have introduced heterogeneity, but our comprehensive subgroup analyses and leave-one-out sensitivity analysis helped identify the main sources of heterogeneity in both outcomes.

In conclusion, our meta-analysis provides evidence that *Lactobacillus* supplementation was associated with a small improvement in *H. pylori* eradication and fewer overall adverse events. However, the optimal Lactobacillus strain, dose, formulation, timing, and duration of supplementation remain uncertain. Future research should adopt standardized probiotic protocols to clarify the optimal supplementation strategy and identify the patient populations most likely to benefit.

## Supplementary Information

Below is the link to the electronic supplementary material.

Supplementary Material 1 (DOCX 8.79 MB)

## Data Availability

No datasets were generated or analysed during the current study.

## Abbreviations

AE: adverse event
CFU: colony–forming units
CI: confidence interval
GRADE: Grading of Recommendations, Assessment, Development, and Evaluation
PPI: Proton pump inhibitor
PRISMA: Preferred Reporting Items for Systematic Reviews and Meta–Analysis
PROSPERO: International Prospective Register of Systematic Reviews
RCT: randomized controlled trial
RoB2: Cochrane Risk of Bias 2
RR: risk ratio
NNT: number needed to treat
RD: risk difference
