# Supplementary material for: Effectiveness of Lactobacillus-supplemented eradication therapy for Helicobacter pylori: a systematic review and meta-analysis of randomized controlled trials
Source: Eur J Clin Pharmacol. 2026 Sep 26;82(10):258. doi: 10.1007/s00228-026-04186-5 (PMC13615058; doi:10.1007/s00228-026-04186-5)
Supplement: Supplementary file 1 [file 228_2026_4186_MOESM1_ESM.docx]

**European Journal of Clinical Pharmacology**

**SUPPLEMENTARY MATERIAL**

**Effectiveness of Lactobacillus-Supplemented Eradication Therapy for Helicobacter pylori: A Systematic Review and Meta-Analysis of Randomized Controlled Trials**

Authors: Karollyna M Henry Moore;^1^ Paula Santo, MD;^2^ Erica Uchoa Holanda, MD;^3^ Angélica Luciana Nau, MD;^4^ Gilmara Coelho Meine, MD, PhD.^5^

^1^ Fundación H. A. Barceló, Buenos Aires, Argentina

^2^ Texas Tech University Health Sciences Center, Lubbock, TX, USA

^3^ University Center Christus, Fortaleza, Brazil

^4^ Department of Pediatric Gastroenterology, Jaraguá Hospital, Jaraguá do Sul, Brazil

^5^ Division of Gastroenterology, Department of Internal Medicine, Feevale University, Novo Hamburgo, Brazil

Corresponding author: Gilmara Coelho Meine

Email: [gilmara@feevale.br](mailto:gilmara@feevale.br)

**Supplementary Table S1.** Search strategy.

| **Database** | **Search strategy** | **Results** |
| --- | --- | --- |
| PubMed | ("Helicobacter pylori" OR "H. pylori") AND (eradication OR therapy OR antibiotic* OR antimicrobial) AND (Lactobacillus OR probiotic*) AND ("randomized controlled trial"[pt] OR "controlled clinical trial"[pt] OR randomized[tiab] OR placebo[tiab] OR "drug therapy"[sh] OR randomly[tiab] OR trial[tiab] OR groups[tiab]) | 536 |
| Embase | ("Helicobacter pylori" OR "H. pylori") AND (eradication OR therapy OR antibiotic* OR antimicrobial) AND (Lactobacillus OR probiotic*) AND ('randomized controlled trial'/exp OR 'controlled clinical trial'/exp OR randomized:ti,ab OR placebo:ti,ab OR 'drug therapy':lnk OR randomly:ti,ab OR trial:ti,ab OR groups:ti,ab) | 1,177 |
| Cochrane | ("Helicobacter pylori" OR "H. pylori") AND (eradication OR therapy OR antibiotic* OR antimicrobial) AND (Lactobacillus OR probiotic*) | 440 |

**Supplementary Table S2.** List of reports excluded at the full-text/detailed eligibility assessment stage and reasons for exclusion.

| First author, year [ref] | Reason for exclusion |
| --- | --- |
| Ameen AM, 2019 [1] | Ineligible probiotic species |
| Hamzavi Y, 2023 [2] | Ineligible probiotic species |
| Pantoflickova D, 2003 [3] | Probiotic monotherapy rather than *Lactobacillus* used as an adjunct to *H. pylori* eradication therapy |
| Noorbakhsh N, 2022 [4] | Ineligible probiotic species |
| Mihai C, 2019 [5] | Additional active co-intervention prevented isolation of the *Lactobacillus* effect |
| Jiang L, 2018 [6] | Ineligible probiotic species |
| FitzGerald J, 2022 [7] | Ineligible probiotic species |
| Hong Q, 2023 [8] | Probiotic monotherapy rather than *Lactobacillus* used as an adjunct to *H. pylori* eradication therapy |
| Tang B, 2021 [9] | Ineligible probiotic species |
| Kakiuchi T, 2020 [10] | Ineligible probiotic species |
| McNicholl AG, 2018 [11] | Ineligible probiotic species |
| Shafaghi A, 2016 [12] | Ineligible probiotic species |
| Francavilla R, 2008 [13] | Probiotic monotherapy rather than *Lactobacillus* used as an adjunct to *H. pylori* eradication therapy |
| Iakovenko EP, 2006 [14] | Non-randomized study design |
| Buckley M, 2018 [15] | Probiotic monotherapy rather than *Lactobacillus* used as an adjunct to *H. pylori* eradication therapy |
| Imase K, 2007 [16] | Probiotic monotherapy rather than *Lactobacillus* used as an adjunct to *H. pylori* eradication therapy |
| Scaccianoce G, 2008 [17] | Ineligible probiotic species |
| Ji W, 2018 [18] | Study protocol; no eligible trial outcome data |
| Du YQ, 2012 [19] | Ineligible probiotic species |
| Haghdoost M, 2017 [20] | Ineligible probiotic species |
| Şirvan BN, 2017 [21] | Ineligible probiotic species |
| Zheng PX, 2016 [22] | In vitro study |
| Naghibzadeh N, 2022 [23] | Ineligible probiotic species |
| Wu Y, 2022 [24] | Ineligible probiotic species |
| Saha BN, 2024 [25] | Ineligible probiotic species |
| Han Z, 2024 [26] | Ineligible probiotic species |
| Azad H, 2024 [27] | Ineligible probiotic species |
| Yan Y, 2024 [28] | Ineligible probiotic species |
| Wang H, 2024 [29] | Additional active co-intervention prevented isolation of the *Lactobacillus* effect |
| Zhang Y, 2024 [30] | Ineligible probiotic species |
| Tokunaga E, 2025 [31] | Ineligible probiotic species |
| Wei W, 2026 [32] | Ineligible probiotic species |
| Yang Y, 2026 [33] | Ineligible probiotic species |
| Cai H, 2023 [34] | Ineligible probiotic species |

**Supplementary Table S3.** Lactobacillus intervention characteristics

| **First author, year** | **Lactobacillus species / strain** | **Formulation** | **Dose** | **Duration, days** | **Timing relative to eradication therapy** |
| --- | --- | --- | --- | --- | --- |
| Armuzzi, 2001 (APT) | *L. rhamnosus* GG (LGG) | Freeze-dried powder | 1.2 × 10¹⁰ CFU/day | 14 | During 7-d therapy + 7 d after |
| Armuzzi, 2001 (Digestion) | *L. rhamnosus GG (LGG)* | Freeze-dried powder | 1.2 × 10¹⁰/day | 14 | During 7-d therapy + 7 d after |
| Canducci, 2000 | Inactivated *L. acidophilus* strain LB | Capsule | 1.5 × 10¹⁰/day | 10 | During 7-d therapy + 3 d after |
| Cremonini, 2002 | *L. rhamnosus* GG (LGG) | Sachet | 1.2 × 10¹⁰ CFU/day | 14 | During 7-d therapy + 7 d after |
| da Silva Medeiros, 2011 | *L. acidophilus* | Capsule | 2.5 × 10¹⁰/day | 8 | During 8-d therapy |
| Deguchi, 2012 | *L. gasseri* OLL2716 | Yogurt | ≥1 × 10⁹ CFU per 112-g serving | 28 | 21 d before + 7 d during therapy |
| Dore, 2019 | *L. reuteri* DSM 17938 + ATCC PTA 6475 | Tablet | 4 × 10⁸ CFU/day | 10 | During 10-d therapy |
| Emara, 2014 | *L. reuteri* DSM 17938 + ATCC PTA 6475 | Chewable tablet | 2 × 10⁸ CFU/day | 28 | During 14-d therapy + 14 d after |
| Francavilla, 2014 | *L. reuteri* DSM 17938 + ATCC PTA 6475 | Chewable tablet | ≥2 × 10⁸ CFU/day | 96 | 28 d before + 7 d during + 61 d after |
| Fu, 2026 | *Lactiplantibacillus plantarum* Lp05 | N/A | 1 × 10¹¹ CFU/day | 42 | During 14-d therapy + 28 d after |
| Han, 2025 | *Lactiplantibacillus plantarum* MH-301 | Packet | >1 × 10⁹ CFU/day | 14 | During 14-d therapy |
| Ismail, 2023 | *L. reuteri* DSM 17648 | Powder-filled capsule | 4 × 10⁹ CFU/day | 30 | During 14-d therapy + 16 d after |
| Ivashkin, 2024 | Inactivated *Limosilactobacillus reuteri* DSM 17648 | Capsule | 4 × 10¹⁰ cells/day | 28 | During 14-d therapy + 14 d after |
| Kamani, 2023 | *Lactobacillus reuteri* | Capsule | N/A | 14 | During 14-d therapy |
| Kiattiweerasak, 2023 | *Lacticaseibacillus rhamnosus* R0011 +  *L. helveticus* R0052 | Capsule | 8 × 10⁹ CFU/day | 42 | During 14-d therapy + 28 d after |
| Lionetti, 2006 | *L. reuteri* ATCC 55730 | Tablet | 1 × 10⁸ CFU qd | 20 | During 10-d therapy + 10 d after |
| Ma, 2015 | *L. acidophilus* | Capsule | N/A | 7 | During 7-d therapy |
| Mohtasham, 2023 | *L. reuteri* | Tablet | N/A | 14 | During 14-d therapy |
| Moreno Márquez, 2022 | *L. reuteri* DSM 17938 + ATCC PTA 6475 | Chewable tablet | N/A | 30 | During 10-d therapy + 20 d after |
| Niu, 2024 | *Lacticaseibacillus rhamnosus* LRa05 | Powder packet | 1 × 10¹⁰ CFU/day | 28 | During 14-d therapy + 14 d after |
| Ojetti, 2012 | *L. reuteri* ATCC 55730 | N/A | 3 × 10⁸ CFU/day | 14 | During 7-d therapy + 7 d after |
| Paoluzi, 2015 | *L. casei DG* | N/A | N/A | 7 | During 7-d therapy |
| Parth, 2021 | Non-viable *L. reuteri* DSM 17648 | Capsule | 2 × 10¹⁰ cells/day | 14 | During 14-d therapy |
| Poonyam, 2019 (A) | *L. reuteri* DSM 17938 + ATCC PTA 6475 | Tablet | N/A | 7 | During 7-d therapy |
| Poonyam, 2019 (B) | *L. reuteri* DSM 17938 + ATCC PTA 6475 | Tablet | N/A | 14 | During 14-d therapy |
| Sahagún-Flores, 2007 | *L. casei* Shirota | N/A | 2.4 × 10¹⁰/day | 7 | During 7-d therapy |
| Shahraki, 2017 | *L. reuteri* Protectis | Tablet | N/A | 28 | During 14-d therapy + 14 d after |
| Sýkora, 2005 | *L. casei* DN-114 001 | Fermented milk | 1 × 10¹⁰ CFU/day | 14 | During 7-d therapy + 7 d after |
| Szajewska, 2009 | *L. rhamnosus* GG | N/A | 2 × 10⁹ CFU/day | 7 | During 7-d therapy |
| Tursi, 2004 | *L. casei* subsp. casei DG | Capsule | 1.6 × 10¹⁰ cells/day | 15 | During 10-d therapy + 5 d after |
| Yang, 2021 | Non-viable *L. reuteri* DSM 17648 | Solid-powder packet | 4 × 10¹⁰ cells/day | 28 | 14 d before + 14 d therapy |
| Zhao, 2025 | *Limosilactobacillus fermentum* MN-LF23 | Freeze-dried powder bag | 1 × 10¹⁰ CFU/day during therapy; 2 × 10¹⁰ CFU/day afterward | 42 | During 14-d therapy + 28 d after |
| Ziemniak, 2006 | *L. acidophilus* + *L. rhamnosus* | Capsule | N/A | 20 | During 10-d therapy + 10 d after |

CFU, colony-forming units; d, days; N/A, not available.

**Supplementary Table S4.** Leave-one-study-out sensitivity analyses for eradication success.

| Study omitted | Pooled RR (95% CI) | τ² | I² | P |
| --- | --- | --- | --- | --- |
| None (primary analysis) | 1.10 (1.05–1.14) | 0.004 | 35% | <0.00001 |
| Armuzzi 2001 (APT) | 1.10 (1.06–1.14) | 0.004 | 37% | <0.001 |
| Armuzzi 2001 (Digestion) | 1.10 (1.06–1.15) | 0.004 | 37% | <0.001 |
| Canducci 2000 | 1.09 (1.05–1.14) | 0.004 | 34% | <0.001 |
| Cremonini 2002 | 1.10 (1.06–1.14) | 0.004 | 37% | <0.001 |
| da Silva Medeiros 2011 | 1.10 (1.06–1.14) | 0.004 | 37% | <0.001 |
| Deguchi 2012 | 1.09 (1.05–1.14) | 0.004 | 35% | <0.001 |
| Dore 2019 | 1.10 (1.06–1.15) | 0.003 | 32% | <0.001 |
| Emara 2014 | 1.10 (1.05–1.14) | 0.004 | 37% | <0.001 |
| Francavilla 2014 | 1.10 (1.05–1.14) | 0.004 | 37% | <0.001 |
| Fu 2026 | 1.10 (1.06–1.15) | 0.004 | 37% | <0.001 |
| Han 2025 | 1.10 (1.06–1.15) | 0.005 | 37% | <0.001 |
| Ismail 2023 | 1.09 (1.05–1.13) | 0.004 | 32% | <0.001 |
| Ivashkin 2024 | 1.10 (1.05–1.14) | 0.004 | 37% | <0.001 |
| Kamani 2023 | 1.11 (1.07–1.15) | 0.001 | 14% | <0.001 |
| Kiattiweerasak 2023 | 1.09 (1.05–1.14) | 0.004 | 35% | <0.001 |
| Lionetti 2006 | 1.10 (1.05–1.14) | 0.004 | 37% | <0.001 |
| Ma 2015 | 1.09 (1.05–1.13) | 0.003 | 28% | <0.001 |
| Moreno Márquez 2022 | 1.10 (1.05–1.14) | 0.004 | 37% | <0.001 |
| Mohtasham 2023 | 1.10 (1.05–1.15) | 0.005 | 37% | <0.001 |
| Niu 2024 | 1.10 (1.05–1.14) | 0.004 | 37% | <0.001 |
| Ojetti 2012 | 1.09 (1.05–1.14) | 0.004 | 34% | <0.001 |
| Paoluzi 2015 | 1.09 (1.05–1.14) | 0.004 | 34% | <0.001 |
| Parth 2021 | 1.09 (1.05–1.14) | 0.004 | 34% | <0.001 |
| Poonyam 2019 (A and B) | 1.10 (1.06–1.15) | 0.005 | 39% | <0.001 |
| Sahagún-Flores 2007 | 1.10 (1.05–1.14) | 0.004 | 36% | <0.001 |
| Shahraki 2017 | 1.10 (1.05–1.14) | 0.004 | 37% | <0.001 |
| Sýkora 2005 | 1.09 (1.05–1.13) | 0.003 | 30% | <0.001 |
| Szajewska 2009 | 1.10 (1.06–1.14) | 0.004 | 37% | <0.001 |
| Tursi 2004 | 1.10 (1.05–1.14) | 0.004 | 37% | <0.001 |
| Yang 2021 | 1.10 (1.06–1.15) | 0.004 | 34% | <0.001 |
| Zhao 2025 | 1.10 (1.06–1.15) | 0.004 | 35% | <0.001 |
| Ziemniak 2006 | 1.10 (1.05–1.14) | 0.005 | 37% | <0.001 |

**Supplementary Table S5.** Leave-one-study-out sensitivity analyses for adverse events.

| Study omitted | Pooled RR (95% CI) | τ² | I² | P |
| --- | --- | --- | --- | --- |
| None (primary analysis) | 0.70 (0.58–0.84) | 0.029 | 36% | 0.0001 |
| Armuzzi 2001 (APT) | 0.71 (0.58–0.86) | 0.035 | 40% | <0.001 |
| Armuzzi 2001 (Digestion) | 0.69 (0.55–0.86) | 0.045 | 42% | <0.001 |
| Canducci 2000 | 0.69 (0.57–0.84) | 0.034 | 42% | <0.001 |
| Cremonini 2002 | 0.72 (0.62–0.84) | 0.014 | 23% | <0.001 |
| Francavilla 2014 | 0.70 (0.57–0.86) | 0.039 | 41% | <0.001 |
| Ivashkin 2024 | 0.68 (0.55–0.84) | 0.040 | 43% | <0.001 |
| Ma 2015 | 0.72 (0.61–0.85) | 0.019 | 28% | <0.001 |
| Mohtasham 2023 | 0.67 (0.52–0.87) | 0.067 | 41% | 0.002 |
| Paoluzi 2015 | 0.71 (0.59–0.85) | 0.026 | 36% | <0.001 |
| Sýkora 2005 | 0.68 (0.56–0.83) | 0.033 | 40% | <0.001 |
| Szajewska 2009 | 0.67 (0.56–0.79) | 0.019 | 27% | <0.001 |
| Tursi 2004 | 0.71 (0.60–0.86) | 0.025 | 34% | <0.001 |

**Supplementary Table S6.** Meta-regression analyses for eradication success and overall adverse events.

| **Outcome** | **Moderator** | **Intercept estimate (P)** | **Moderator estimate (P)** | **Residual I²** | **Residual heterogeneity P** |
| --- | --- | --- | --- | --- | --- |
| Eradication success | Lactobacillus dosage (log10 CFU/day) | 0.2262 (0.3287) | -0.0140 (0.5577) | 36.87% | 0.0546 |
| Overall adverse events | Lactobacillus dosage (log10 CFU/day) | -0.2509 (0.7441) | 0.0128 (0.1665) | 65.98% | 0.0083 |

**Supplementary Figure S1.** Individual adverse events.

**A. Abdominal pain**

**B. Heartburn**

**C. Nausea**

**D. Diarrhea**

**Supplementary Figure S2.** Subgroup analysis of H. pylori eradication success according to comparator type.

**Supplementary Figure S3.** Subgroup analysis of H. pylori eradication success according to eradication regimen.

**Supplementary Figure S4.** Subgroup analysis of H. pylori eradication success according to duration of eradication therapy.

**Supplementary Figure S5.** Subgroup analysis of H. pylori eradication success according to Lactobacillus strain category.

**Supplementary Figure S6.** Subgroup analysis of H. pylori eradication success according to daily Lactobacillus dose.

**Supplementary Figure S7.** Subgroup analysis of H. pylori eradication success according to timing of Lactobacillus administration relative to eradication therapy.

**Supplementary Figure S8.** Subgroup analysis of H. pylori eradication success according to Lactobacillus formulation.

**Supplementary Figure S9.** Subgroup analysis of H. pylori eradication success according to geographical region.

**Supplementary Figure S10.** Subgroup analysis of H. pylori eradication success according to age group: adults versus children.

**Supplementary Figure S11.** Subgroup analysis of overall adverse events according to comparator type.

.

**Supplementary Figure S12.** Subgroup analysis of overall adverse events according to eradication regimen.

**Supplementary Figure S13.** Subgroup analysis of overall adverse events according to duration of eradication therapy.

**Supplementary Figure S14.** Subgroup analysis of overall adverse events according to Lactobacillus strain category.

**Supplementary Figure S15.** Subgroup analysis of overall adverse events according to timing of Lactobacillus administration relative to eradication therapy.

**Supplementary Figure S16.** Subgroup analysis of overall adverse events according to Lactobacillus formulation.

**Supplementary Figure S17.** Subgroup analysis of overall adverse events according to geographical region.

**Supplementary Figure S18.** Subgroup analysis of overall adverse events according to age group.

**Supplementary Figure S19.** Risk of bias assessment (RoB 2).

**Supplementary Figure S20.** Funnel plot for eradication success.

**Supplementary Figure S21.** Funnel plot for overall adverse events.

**REFERENCES**

1. Ameen AM, Abdulridha MK, Najeeb AA. Comparative effectiveness of probiotics timing regimen in Helicobacter pylori-induced peptic ulcer disease patients. J Pharm Sci Res. 2019;11(1):75–83.

2. Hamzavi Y, Bashiri H. The effect of quadruple therapy plus probiotics on Helicobacter pylori eradication and antibiotic-associated side effects: a randomized placebo-controlled trial. J Kermanshah Univ Med Sci. 2023;27(4).

3. Pantoflickova D, Corthésy-Theulaz I, Blum AL, et al. Favourable effect of regular intake of fermented milk containing Lactobacillus johnsonii on Helicobacter pylori-associated gastritis. Aliment Pharmacol Ther. 2003;18(8):805–813.

4. Noorbakhsh N, Nikpour S, Salehi M. The efficacy and safety of furazolidone-bismuth quadruple therapy for Helicobacter pylori eradication with or without probiotic supplementation. Gastroenterol Hepatol Bed Bench. 2022;15(2):146.

5. Mihai C, Popescu R, Ionescu A, et al. Lactobacillus reuteri – an alternative in the first-line of Helicobacter pylori eradication. Farmacia. 2019;67(5):871–876.

6. Jiang L, Zhu W. Probiotics improved the effectiveness and safety of the quadruple Helicobacter pylori eradication therapy. Biomed Res. 2018;29(10):2053–2056.

7. FitzGerald J, Wang T, Wang L, et al. Improved gut microbiome recovery following drug therapy is linked to abundance and replication of probiotic strains. Gut Microbes. 2022;14(1):2094664.

8. Hong Q, Liu Y, Zhao Y, et al. Study of the effect of Lactobacillus crispatus FSCDJY67L3 on Helicobacter pylori eradication: a double-blind randomized controlled clinical trial. Front Immunol. 2023;14:1265995.

9. Tang B, Tang L, Huang C, et al. The effect of probiotics supplementation on gut microbiota after Helicobacter pylori eradication: a multicenter randomized controlled trial. Infect Dis Ther. 2021;10:317–333.

10. Kakiuchi T, Matsuo K, Kishino T, et al. Effect of probiotics during vonoprazan-containing triple therapy on gut microbiota in Helicobacter pylori infection: a randomized controlled trial. Helicobacter. 2020;25(3):e12690.

11. McNicholl AG, Linares PM, Nyssen OP, et al. Probiotic supplementation with Lactobacillus plantarum and Pediococcus acidilactici for Helicobacter pylori therapy: a randomized, double-blind, placebo-controlled trial. Helicobacter. 2018;23(5):e12529.

12. Shafaghi A, Pourkazemi A, Khosravani M, et al. The effect of probiotic plus prebiotic supplementation on the tolerance and efficacy of Helicobacter pylori eradication quadruple therapy: a randomized prospective double-blind controlled trial. Middle East J Dig Dis. 2016;8(3):179–188.

13. Francavilla R, Lionetti E, Castellaneta SP, et al. Inhibition of Helicobacter pylori infection in humans by Lactobacillus reuteri ATCC 55730 and effect on eradication therapy: a pilot study. Helicobacter. 2008;13(2):127–134.

14. Iakovenko EP, Kravtsova OA, Nikitin AA, et al. Effects of probiotic Bifiform on efficacy of Helicobacter pylori infection treatment. Ter Arkh. 2006;78(2):21–26.

15. Buckley M, Lacey S, Doolan A, et al. The effect of Lactobacillus reuteri supplementation in Helicobacter pylori infection: a placebo-controlled, single-blind study. BMC Nutr. 2018;4:1–8.

16. Imase K, Tanaka A, Tokunaga K, et al. Lactobacillus reuteri tablets suppress Helicobacter pylori infection: a double-blind randomised placebo-controlled cross-over clinical study. Kansenshogaku Zasshi. 2007;81(4):387–393.

17. Scaccianoce G, Zullo A, Hassan C, et al. Triple therapies plus different probiotics for Helicobacter pylori eradication. Eur Rev Med Pharmacol Sci. 2008;12(4):251–256.

18. Ji W, Chen WQ, Tian X. Efficacy of compound Lactobacillus acidophilus tablets combined with quadruple therapy for Helicobacter pylori eradication and its correlation with pH value in the stomach: a study protocol of a randomised, assessor-blinded, single-centre study. BMJ Open. 2018;8(10):e023131.

19. Du YQ, Su T, Lu H, et al. Adjuvant probiotics improve the eradication effect of triple therapy for Helicobacter pylori infection. World J Gastroenterol. 2012;18(43):6302.

20. Haghdoost M, Sadeghi A, Salari M, et al. Double strain probiotic effect on Helicobacter pylori infection treatment: a double-blinded randomized controlled trial. Caspian J Intern Med. 2017;8(3):165.

21. Şirvan BN, Demir H, Aydemir G, et al. Are synbiotics added to the standard therapy to eradicate Helicobacter pylori in children beneficial? A randomized controlled study. EurAsian J Hepatogastroenterol. 2017;7(1):17.

22. Zheng PX, Liu J, Wang Y, et al. Lactobacillus pentosus strain LPS16 produces lactic acid, inhibiting multidrug-resistant Helicobacter pylori. J Microbiol Immunol Infect. 2016;49(2):168–174.

23. Naghibzadeh N, Salmani F, Nomiri S, Tavakoli T. Investigating the effect of quadruple therapy with Saccharomyces boulardii or Lactobacillus reuteri strain DSMZ 17648 supplements on eradication of Helicobacter pylori and treatment’s adverse effects: a double-blind placebo-controlled randomized clinical trial. BMC Gastroenterol. 2022;22(1):107.

24. Wu Y, Lin Y, Zhang Y, et al. Effects of probiotics on gastric microbiota and its precombination with quadruple regimen for Helicobacter pylori eradication. J Dig Dis. 2022;23(8–9):462–472.

25. Saha BN, Sarkar MAM, Hasan MN, et al. Efficacy of probiotics as adjuvant to the standard triple therapy for the treatment of Helicobacter pylori-associated peptic ulcer disease: a randomized-control trial. Mymensingh Med J. 2024;33(4):1166–1171.

26. Han Z, Li Y, Nan X, Zhou T, Li L, Li Y. Effect of probiotic supplementation combined with bismuth-containing quadruple therapy on gut microbiota during Helicobacter pylori eradication: a randomized, double-blind, placebo-controlled trial. Front Nutr. 2024;11:1484646.

27. Azad H, Hormati A, Sarkeshikian SS, et al. Efficacy of Pyloshot in combination with standard four-drug antimicrobial treatment for Helicobacter pylori eradication: a randomized clinical trial. JGH Open. 2024;8(12):e70046.

28. Yan Y, Dong L, Xu J, et al. Preliminary study on the potential impact of probiotic combination therapy on Helicobacter pylori infection in children using 16S gene sequencing and untargeted metabolomics approach. Front Microbiol. 2024;15:1487978.

29. Wang H, Wei W, Liu F, Wang M, Zhang Y, Du S. Effects of fucoidan and synbiotics supplementation during bismuth quadruple therapy of Helicobacter pylori infection on gut microbial homeostasis: an open-label, randomized clinical trial. Front Nutr. 2024;11:1407736.

30. Zhang Y, Lu B, Dong Y, et al. Saccharomyces boulardii combined with triple therapy alter the microbiota in the eradication of Helicobacter pylori infection. Sci Rep. 2024;14(1):13152.

31. Tokunaga E, Inoue J, Asaji N, et al. Impact of Enterococcus faecium 129 BIO 3B-R on Helicobacter pylori eradication therapy side effects in adult patients: a randomized, double-blind, placebo-controlled study. Sci Rep. 2025;15:32085.

32. Wei W, Chen W, Peng R, Wang X, Wang Z, Zhang Z. Probiotics alleviate oral microbiota disruptions induced by Helicobacter pylori eradication with vonoprazan-amoxicillin dual therapy: a randomized double-blind placebo-controlled trial (an exploratory study). Front Cell Infect Microbiol. 2026;16:1740968.

33. Yang Y, Lan C, Jiang Z. Impact of probiotic-adjunctive vonoprazan-amoxicillin dual therapy on gut microbiota and clinical symptoms in Helicobacter pylori eradication. Tissue Eng Regen Med. 2026;23(5):653–666.

34. Cai H, Si X. Lactobacillus acidophilus enhances relief from clinical symptoms of Helicobacter pylori infection in patients on quadruple therapy. Curr Top Nutraceutical Res. 2023;21(2):125-130.
